# Combination of Pre- and Post-Mercerization Processes for Cotton Fabric

**DOI:** 10.3390/ma15062092

**Published:** 2022-03-11

**Authors:** Lina Lin, Tiancheng Jiang, Yonghong Liang, Wenju Zhu, Umarsharif Y. Inamdar, Md. Nahid Pervez, Rahul Navik, Xiaojun Yang, Yingjie Cai, Vincenzo Naddeo

**Affiliations:** 1 Hubei Provincial Engineering Laboratory for Clean Production and High-Value Utilization of Bio-Based Textile Materials, Wuhan Textile University, Wuhan 430200, China; linalin@wtu.edu.cn (L.L.); 2015383113@mail.wtu.edu.cn (T.J.); 1815073028@mail.wtu.edu.cn (Y.L.); 1915223024@mail.wtu.edu.cn (W.Z.); umar.inamdar@yandex.com (U.Y.I.); mpervez@unisa.it (M.N.P.); 2 Engineering Research Centre for Clean Production of Textile Dyeing and Printing, Ministry of Education, Wuhan Textile University, Wuhan 430200, China; 3 Sanitary Environmental Engineering Division (SEED), Department of Civil Engineering, University of Salerno, 84084 Fisciano, Italy; 4 School of Chemistry and Chemical Engineering, Shanghai Jiao Tong University, Shanghai 200240, China; rahul.navik2012@gmail.com

**Keywords:** pre-mercerization, post-mercerization, combined mercerization, woven cotton fabric, stiffness

## Abstract

The dyeing process commonly deteriorates the luster of pre-mercerized cotton fabric, so post-mercerization processes are regularly applied to compensate for this. Herein, the influence of combining pre-mercerization with CS (caustic solution) or LA (liquid ammonia) and post-mercerization with CS or LA on the morphological structure, dyeing performance, tensile strength, and stiffness of woven cotton fabric was investigated. The crystallinity index values greatly decreased from 73.12 to 51.25, 58.73, 38.42, and 40.90% after the combined mercerization processes of LA–LA, CS–CS, LA–CS, and CS–LA, respectively. Additionally, the CS–LA- and LA–CS-treated samples exhibited a mixture of cellulose II and cellulose III allomorphs. The combined mercerization processing of cotton fabric resulted in slightly worse thermal stability. The LA and CS pre-mercerization processes increased the dye exhaustion, although the former decreased the dye fixation rate while the latter increased it by 4% for both dyes. The color strength of the dyed cotton fabric increased after both post-mercerization processes. Moreover, the fabric stiffness and mechanical properties showed an increasing trend due to the combined mercerization efforts.

## 1. Introduction

Cotton fiber is popularly used in the production of apparel because of its inherent hydrophilicity, comfort, dyeability, flexibility, and breathability [1,2,3,4]. However, cotton fiber requires tedious modification processes, such as ultraviolet irradiation [5], ultrasound [6], microwave irradiation [7], gamma irradiation [8], and plasma treatment [9] to overcome the limited surface wettability, functional characteristics, and dyeing performance. However, these processes are costly, environmentally unpleasant, and lack scale-up feasibility. Thus, customization of the conventional mercerization process through various approaches can be an interesting choice to improve the characteristics of cotton fiber.

Mercerization treatment by either strong caustic solution (CS) or liquid ammonia (LA) with tension has an attractive effect on the dyeability of cotton fiber and the luster of the resulting fabric [10,11]. Through mercerization, the crystallinity of the cotton fiber decreases and the amorphous characteristics increase, producing more pores and effective hydroxyl groups in the fiber, which is beneficial for dye exhaustion [12] and fixation [13,14], leading to improved dyeability [10]. Thus, pre-mercerization (mercerization before dyeing) is usually selected to enhance cotton dyeing properties.

Post-mercerization (mercerization after dyeing) is preferentially applied to produce a luster for dyed cotton fabric to meet special requirements. However, post-mercerization causes a color shift due to the hydrolysis of the covalent bond (ether bond) between the reactive group of the reactive dye and the hydroxyl group of the cotton fiber for the morphological structure transformation of the cotton fiber [15]. Moreover, the morphological structure transformation also alters the mechanical properties and hand feel of dyed cotton fabric [15]. Thus, this indicates that morphological structure transformation is an important factor contributing to mercerization performance. Since the morphological structure transformation effectively contributes to the mercerization performance of cotton fabric, the morphological differences between the dyed cotton fabric with and without pre-mercerization may result in different post-mercerization performances, such as differences in the color shade, mechanical properties, hand feel, and so on, which is a research gap regarding the mercerization treatment of cellulosic fabric.

Sometimes, the luster of cotton fabric produced by pre-mercerization is lessened by the subsequent dyeing process, so post-mercerization is used to compensate for this loss in luster. However, there are no reports regarding the combination of pre- and post- mercerization for cotton fabric. Thus, pre-mercerization by CS or LA, dyeing with reactive dye, and post-mercerization of cotton fabric by CS or LA were investigated.

The driving forces of this work are to explain the contradictory phenomenon whereby LA pre-mercerization of cotton fabric is occasionally ineffective in promoting cotton dyeability and to clarify the influence of the morphological structure of cotton fiber on CS or LA post-mercerization, including the morphological structure, mechanical properties, and color shade. These research achievements are significant in guiding and instructing mercerization treatment, especially for the combination of pre- and post-mercerization for cotton fabric.

## 2. Materials and Methods

### 2.1. Materials

Commercially scoured and bleached, 100% plain woven cotton fabric (160 g m^−2^ GSM, 0.39 mm thickness, 1/1 repeat unit cell) was received from the TST Group Holding, Ltd. (Guangzhou, China). Two commercial reactive dyes, C. I. Reactive Red 2 (R2) and C. I. Reactive Red 195 (R195), were supplied by Shanghai Jiaying Chemical Co. Ltd. (Shanghai, China) and directly used in the dyeing process. The detailed properties of these two dyes are listed in Table 1. Liquid ammonia (purity of 99.99%) was purchased from Wuhan Niuruide Gas Co., Ltd. (Wuhan, China). Non-ionic detergent (Luton 500) was purchased from Dalton UK Company (Shanghai, China). All other chemicals and reagents used in this work were of analytical grade.

Diverse cotton fabrics were prepared and analyzed, including O-Dye (original dyed cotton fabric), CS-Dye (CS pre-mercerized and dyed cotton fabric), LA-Dye (LA pre-mercerized and dyed cotton fabric), Dye-CS (CS post-mercerized and dyed cotton fabric that without pre-mercerization), Dye-LA (LA post-mercerized and dyed cotton fabric that without pre-mercerization), CS-Dye-CS (CS post-mercerized and dyed cotton fabric with CS pre-mercerization), CS-Dye-LA (LA post-mercerized dyed cotton fabric with CS pre-mercerization), LA-Dye-CS (CS post-mercerized dyed cotton fabric with LA pre-mercerization), and LA-Dye-LA (LA post-mercerized dyed cotton fabric with LA pre-mercerization).

### 2.2. Mercerization

#### 2.2.1. Caustic Mercerization

For pre-mercerization, fabric under tension was immersed in 250 g L^−1^ NaOH solution at room temperature (23 °C) for 3 min and then washed twice in hot water at 60–70 °C for 5 min, followed by washing in water with 2 g L^−1^ HCl at room temperature for 5 min. Finally, it was rinsed with tap water until neutral. While, for post-mercerization, after treatment, the post-mercerized and dyed cotton fabric was cleaned with a 2 g L^−1^ of non-ionic detergent solution for 30 min at 95 °C with a liquor ratio of 100:1.

#### 2.2.2. Liquid Ammonia Mercerization

For pre-mercerization, fabric under tension was immersed in anhydrous liquid ammonia at −40 °C for 3 min, followed by drying in an oven at 60 °C for 10 min. Subsequently, the dried fabric was rinsed with tap water until neutral. For post-mercerization, after treatment, the post-mercerized and dyed cotton fabric was cleaned with a 2 g L^−1^ of non-ionic detergent solution for 30 min at 95 °C with a liquor ratio of 100:1.

### 2.3. Dyeing Performance

#### 2.3.1. Dyeing Process of Cotton Fabrics

**Figure 1:**
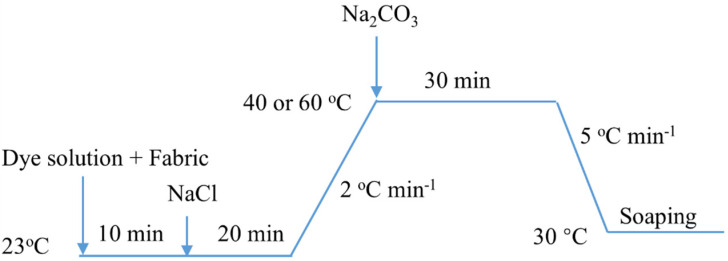
Dyeing of cotton fabric with R2 or R195.

The dyeing process (Figure 1) was carried out using a laboratory infrared dyeing machine (HB-HW × 24, Huibao, Foshan, China). The fabric was dyed with R2 at 40 °C or R195 at 60 °C at a liquor ratio of 20:1 using 3% o.m.f (on the mass of fiber) with 40 g L^−1^ of NaCl and 10 g L^−1^ of Na_2_CO_3_. After dyeing, the dyed fabric was cleaned in a solution containing 2 g L^−1^ non-ionic detergent for 30 min at 95 °C using a liquor ratio of 50:1.

#### 2.3.2. Measurement of Dyeing Performance

The exhaustion percentage (*E*%) of dye can be defined as the mass of dye adsorbed onto the fabric after the dyeing process (Equation (1)) [18,19], where A_0_ is the absorbance of dye solution before dyeing and A_1_ indicates the residual dye solution absorbance after dyeing. Next, the dye fixation rate (*F*%) was determined by measuring the mass of dye fixed in the fabric, followed by the soaping process with the mass of dye adsorbed after exhaustion dyeing onto the fabric (Equation (2)), where A_2_ is the absorbance of the soaped solution. The total dye fixation efficiency (*T*%) was used to represent the total efficiency of dye mass application by measuring the mass of dye fixed in the fabric after the soaping stage with an initial amount of dye applied (Equation (3)). The absorbance value of the dye solution was assessed at the maximum wavelength for R2 (540 nm) and R195 (542 nm) using a Cary 100 UV–visible spectrophotometer (Agilent Technologies, Melbourne, Australia).

(1) $$E\%=\frac{A_{0}-A_{1}}{A_{0}}\;\times \;100\%,$$

(2) $$F\%=\frac{A_{0}-A_{1}-A_{2}}{A_{0}-A_{1}}\;\times \;100\%,$$

(3) T%=E% × F% × 100,

#### 2.3.3. Color Strength and Color Uniformity

The color strength of the dyed fabric sample (expressed by *K/S* value) was measured using a laboratory spectrophotometer (Datacolor 110, Datacolor International, Rotkreuz, Switzerland). Twenty places were randomly selected for measuring and average values were reported. The standard deviations of these 20 *K/S* values were used to evaluate the color uniformity of dyed fabric, and the color uniformity of the dyed sample was higher when the value of the standard deviation was lower [20]. Moreover, the average value of 20 *K/S* values was used to calculate the promoted *K/S* efficiency (*P*%) via mercerization using Equation (4):

(4) $$P\%=\frac{{\left(K/S\right)}_{1}-{\left(K/S\right)}_{0}}{{\left(K/S\right)}_{0}}\times 100\%,$$

where (*K/S*)_0_ is a predicted value that represents the original dyed fabric’s predicted *K/S* value, which has the same dye concentration as the pre-mercerized and dyed fabric; (*K/S*)_1_ is an experimental value that represents the pre-mercerized and dyed fabric’s *K/S* value.

#### 2.3.4. Colorfastness to Washing and Rubbing

Colorfastness to washing was measured in accordance with ISO 105-C06:2010 “Textiles—Tests for Colorfastness—Part C06: Colorfastness to Domestic and Commercial Laundering” (test number: C2S). Colorfastness to washing is graded by measuring the staining on cotton fiber of a multi-fiber strip and comparing it with ISO grayscale standard. The colorfastness to rubbing was evaluated using a crock meter by following the method of ISO 105-X12:2016 “Textiles—Tests for Colorfastness—Part X12: Colorfastness to Rubbing”.

#### 2.3.5. Dye Removal Percentage by CS and LA Post-Mercerization Processes

After post-mercerization, the light absorbance of the residual soaped solution was measured and the dye removal percentage was calculated using Equation (5):

(5) $$R\%=\frac{A_{3}}{\left(A_{0}-A_{1}-A_{2}\right)}\times 100\%,$$

where *A*_0_ and *A*_1_ represent the dye solution absorbance value before and after the dyeing process, respectively. *A*_2_ is the residual soaping solution absorbance value and *A*_3_ is the residual washing solution absorbance of both CS- or LA-mercerized dyed fabric.

### 2.4. Measurements and Characterization

#### 2.4.1. X-ray Diffraction

X-ray diffraction (XRD) analysis was performed for the cotton fabrics using a powder X-ray diffractometer (Rigaku Ultima III, Tokyo, Japan). Before measurements, the samples were converted into a fine powder using a scissor. Afterwards, about 0.15 g of powder was pressed through the use of a hydraulic press at 127 MPa using a circular disk. The diameter was then converted into a 2.5-cm-diameter scale. The samples were placed onto a holder and scan parameters ranged from 2θ values of 5° to 50° with a 2θ step of 0.02° under CuKα radiation (λ = 1.54056 Ǻ).

#### 2.4.2. Thermogravimetric Analysis

To investigate the thermal properties of the samples, a thermogravimetric (TG) analyzer (TGA/DSC1, Mettler-Toledo, LLC, Shanghai, China) was used, which maintained heating rates at around 10 °C min^−1^ over a range of 30 to 700 °C. TG analysis was performed at a flow rate of 50 mL min^−1^ under nitrogen.

#### 2.4.3. Stiffness

The stiffness properties of the woven cotton fabrics were characterized by their bending behavior, which was determined according to GB/T 18318.1-2009 “Textiles—Determination of Bending Behavior—Part 1: Incline method”.

#### 2.4.4. Breaking Force and Elongation of Fabrics

The breaking force and elongation at break were detected according to the standard method of GB/T 3923.1-1997 “Textiles—Tensile Properties of Fabrics—Part 1: Determination of Breaking Force and Elongation at Breaking Force—Strip Method”.

## 3. Results and Discussion

### 3.1. XRD Analysis

After different mercerization processes, changes in crystallinity and cellulose allomorphs of each sample were investigated using XRD patterns. The FitYK 1.3.1 program was used to subtract the background and divide the diffraction peaks. In order to calculate the crystalline index (CI) of the samples, Equation (6) was used to calculate the area occupied by the diffraction peaks in each sample.

(6) $$CI\%=\frac{I_{c}}{I_{c}+I_{a}}\times 100\%,$$

where *I*_c_ represents the crystalline phase integrated intensity and *I*_a_ is the amorphous phase-integrated intensity.

The values of CI for the original and mercerized samples are summarized in Table 2. The CI values decreased from 71.52% (original) to 52.61% and 63.19% after LA and CS mercerization processes, respectively. The CI value further decreased to 51.25% and 58.73% after LA–LA and CS–CS mercerization processes. The patterns recorded from LA–CS and CS–LA-treated cotton consisted of 38.42% and 40.90% CI, respectively. The decreased CI values of the samples suggest that these mercerization processes, either individually or in combination, resulted in micro-fibril swelling, rearrangement of compact hydrogen bonds in crystalline regions leading to crystallite disruption, and the formation of new amorphous regions. Treatments with combined mercerization processes resulted in a greater degree of crystallite disruption, which exhibited an advantage with the rearrangement of hydrogen bonding to cellulose crystals and the generation of a high-volume amorphous region. These results can mainly be attributed to the collaborative mercerization effect by LA and CS.

**Figure 2:**
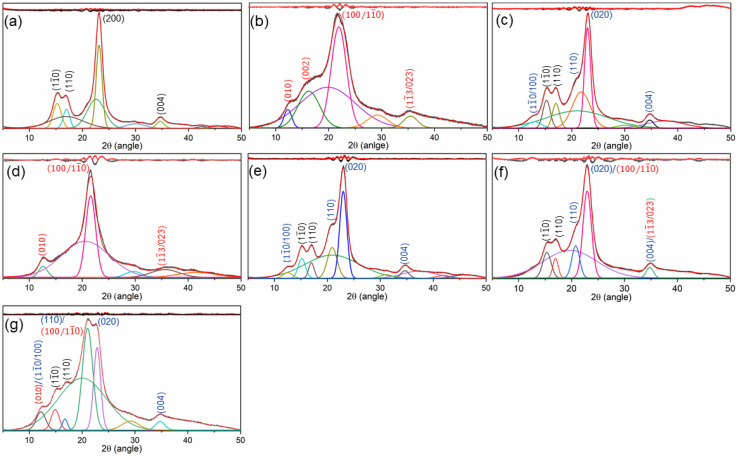
XRD images of the (**a**) original, (**b**) LA, (**c**) CS, (**d**) LA–LA, (**e**) CS–CS, (**f**) LA–CS, and (**g**) CS–LA-mercerized cotton samples.

Besides the changes in *CI*, the cellulose allomorph conversion by these mercerization processes was also investigated. Figure 2 compares the diffraction patterns of the original and mercerized cotton fabrics. As shown in Figure 2a, the diffraction pattern of original cotton displayed obvious characteristics of the cellulose I allomorph, consisting of peaks at 2θ values of about 14.7, 16.8, 22.6, and 34.8°, which were attributed to (1$\bar{1}0$), (110), (200), and (004) lattice planes, respectively [21,22,23]. In addition, the peaks were sharper, which suggests that large crystals were present in the cellulose phase [24]. There was also a significant shift in diffraction patterns following LA- and CS-mercerized cotton fabrics (Figure 2b–g). The LA pre-mercerization and combined LA–LA mercerization processes led to the formation of a diffraction pattern of cellulose III allomorph consisting of (010), (011), (100/1$\bar{1}$0), and (013/022) lattice planes at 2θ values of 11.7, 15.5, 21.0, and 35.0°, respectively [21,25]. Using CS pre-mercerization as well as the combined CS–CS mercerization processes, cellulose I was converted to a cellulose II allomorph, with the lattice planes of (1$\bar{1}$0/100), (110), (020), and 004 being found at 2θ values of 12.2, 20.2, 21.8, and 34.7°, respectively [26,27]. Interestingly, with the combined LA–CS and CS–LA mercerization processes, the obtained diffraction pattern revealed the presence of both cellulose II and cellulose III allomorphs in the cotton.

The patterns exhibited (1$\bar{1}$0/100), (110), (020), and (004) lattice planes of cellulose II and (010) and (100/$1\bar{1}$0) lattice planes of cellulose III. Cellulose allomorph conversion occurred as a result of the appearance of a new crystalline domain in the mercerized cotton fibers. Briefly, in cellulose microfibrils, NH_3_ and NaOH systems penetrated through the amorphous and crystalline regions to result in fiber swelling and cellulose I transformation into Na–cellulose and NH_3_–cellulose. The Na–cellulose I complex was converted into Na–cellulose II, while the complex NH_3_–cellulose I was turned into NH_3_–cellulose III. However, new hydrogen bonding networks with completely different patterns and cross-binding patterns, named cellulose II and cellulose III, were generated with the removal of NaOH and NH_3_ from these cellulose complexes in the amorphous and crystalline regions [11,28]. Besides this, the cellulose microfibrils led to difficult recrystallization and the treated samples were mainly composed of smaller crystallites within the fibers [29].

### 3.2. Thermal Performance

**Figure 3:**
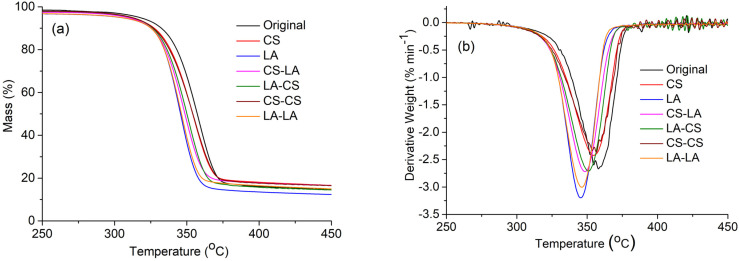
(**a**) TG thermograms and (**b**) DTG curves of original and mercerized cotton fabrics.

The thermal stability of a cotton fabric is an essential property with respect to its practical applications. Therefore, the thermal behaviors of original and mercerized fabrics with different conditions were thoroughly investigated using TG analysis. As shown in Figure 3a, the TG thermogram patterns were similar for both the original and mercerized fabrics. However, slight variations were noticed with respect to the weight loss percentage for each sample at various temperatures and their char residue amounts (Table 3). It was shown that the initial cleavage onset temperature was higher (T_onset_, 321 °C) for the original fabric than the mercerized fabrics, which means that the mercerized samples decomposed much more rapidly than the original sample. The maximum weight loss began at 372 °C for the original fabric because of the depolymerization behavior mediated by trans-glycosylation [30]. Meanwhile, the maximum weight loss temperatures (T_max_) decreased to 370 and 361 °C for CS- and LA-mercerized fabrics, respectively, and for the combined mercerization processes, these values were significantly lower. These results can be explained by the higher amount of amorphous cellulose in the combined mercerization samples, meaning they underwent faster thermal decomposition than the crystalline phase [31].

In addition, as shown the char amounts were 10.80% for the original fabric, 10.73% for CS-mercerized fabric, and 8.18% for the LA-mercerized fabric. Moreover, the highest char amount for the combination of CS–CS-mercerized fabric was 12.31%, and in the case of combined LA–LA mercerization, the char amount was less (10.01%). These phenomena were further verified from the DTG curves (Figure 3b), which were the derivatives of the TG curves. Overall, the data suggests that LA-mercerized fabric has slightly less thermal stability than CS-mercerized fabric, in agreement with a previous report [25].

### 3.3. Influence of Pre-Mercerization on Dyeing Performance of Cotton Fabric

**Figure 4:**
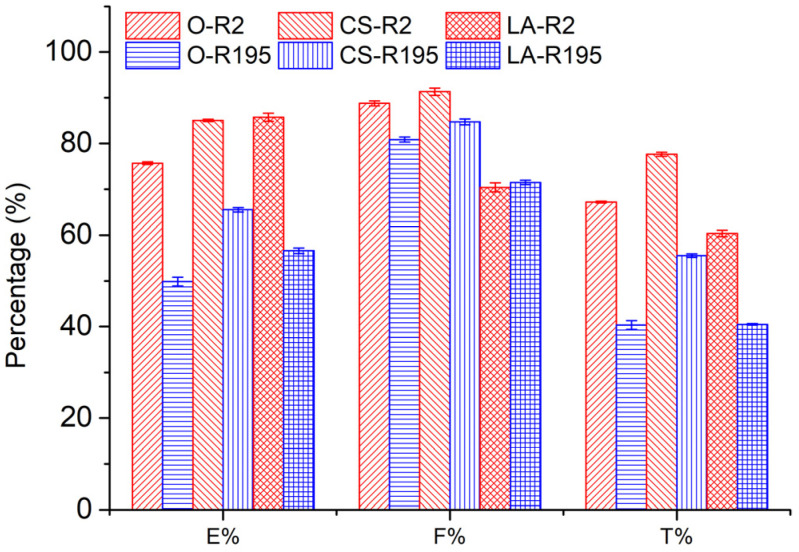
Dyeing performance of woven cotton fabric pre-mercerized by CS and LA using R2 and R195 dyes.

The *E*%, *F*%, and *T*% values after dyeing original and CS and LA pre-mercerized cotton fabrics using R2 and R195 are shown in Figure 4. It is obvious that CS and LA pre-mercerization processes enhanced the *E*% values of cotton fabrics after dyeing, which is consistent with the results of a previous report [14]. During dyeing, dye molecules are only located in the amorphous region of the cotton fiber; thus, a more crystalline cotton fiber usually exhibits lower dye exhaustion. Both CS and LA pre-mercerization processes partially damaged the crystallinity, which resulted in a decreased crystalline region and increased amorphous region. Therefore, the cotton fabrics pre-mercerized by CS or LA showed a higher *E*% level.

In the dyeing of cotton fiber, dye adsorption is achieved in the amorphous area through dye migration from the surface of the fiber via pores, and a small pore size allows small-molecule transfer but impedes larger molecule migration [25]. After both CS and LA pre-mercerization processes, the cumulative accessible pore volume of cotton fiber increased [32], as identified by the decreased CI value; however, the former enlarged the pore size while the latter contracted it [33]. In dyeing with R2, the CS and LA pre-mercerization processes contributed a similar increment to the *E*% of cotton fabric dyeing, of which the *E*% values were 85.1% and 85.7%, respectively. However, in dyeing with R195, CS pre-mercerization promoted an apparently higher *E*% than with LA pre-mercerization, where the *E*% values were 65.5 and 56.6% for the CS and LA pre-mercerization processes, respectively. This result indicated that the *E*% was influenced by the molecular size of the dye (molecular weight) and the pore size of the cotton fiber, which can be seen by the molecular size of R2 (molecular weight: 615.32 g mol^−1^, Table 1) showing a smaller size than that of R195 (molecular weight: 1136.28 g mol^−1^, Table 1).

In dye fixing, the reactive group of the reactive dye forms a covalent bond with the hydroxyl group of the cotton fiber. In contrast with the original cotton fibers, the *F*% values of CS pre-mercerized cotton fabrics dyed using R2 and R195 were promoted slightly, but the *F*% values decreased in the dyeing of LA pre-mercerized cotton fabric using R2 and R195 dyes, although more hydroxyl groups in the cotton fiber were produced due to the crystallinity damage caused by CS and LA pre-mercerization processes. R195 has bifunctional reactive groups and R2 has a dichlorotriazinyl group, although the *F*% values did not show the superiority of R195 in dye fixing, which was possibly due to the more active dichlorotriazinyl group facilitating the formation of the covalent bond with the cellulosate under fixation conditions.

In the dye fixation stage, after the addition of soda ash, the dye fixing reaction is expedited, accompanied by the deterioration of the dye adsorption balance between the dye in the fiber and dye bath, resulting in further transference of the dye from the dye bath to the fiber, i.e., dye exhaustion and fixation simultaneously occurred. Therefore, dye fixation also contributed to the *E*% increase, which was one factor of the higher *E*% values for R2 dyeing compared to R195 dyeing.

*T*% is dependent on *E*% and *F*%, so the increases in *E*% and *F*% by CS pre-mercerization caused the *T*% values of CS pre-mercerized cotton fabrics dyed using R2 and R195 to be higher than that of the original and LA pre-mercerized cotton fabrics. In LA pre-mercerized cotton fabrics, the *T*% values of fabrics dyed using R2 and R195 decreased in comparison with original fabrics. However, when comparing *T*% values, the R2 dyeing process exhibited a higher value than R195 dyeing, which was due to the higher *E*%. In addition, the standard deviation values of *E*%, *F*%, and *T*% (error bars in Figure 4) were smaller, which indicated that the dyeing performance was repeatable.

### 3.4. Influence of Post-Mercerization on Dye Removal

**Figure 5:**
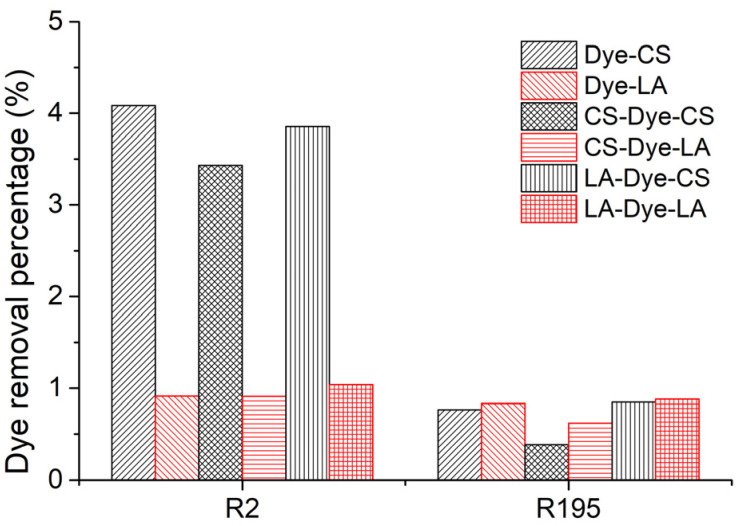
Effects of CS and LA post-mercerization on the dye stability of dyed woven cotton fabrics using R2 and R195 dyes.

**Figure 6:**
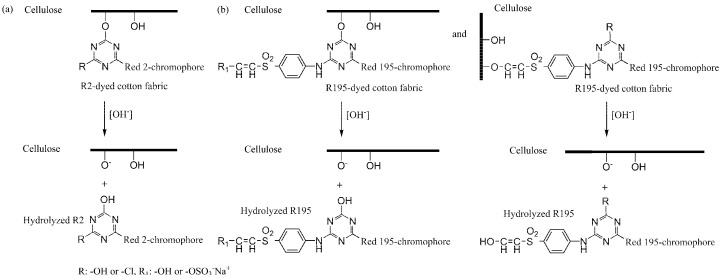
Hydrolysis of the ether bonds between the cellulose and reactive dyes of (**a**) R2 and (**b**) R195.

In CS and LA post-mercerization of dyed cotton fabrics, dye stability is a significant factor. The results of these processes are displayed in Figure 5. After post-mercerization, we attempted to wash the R2 and R195 dyes from the fabrics. In contrast with CS post-mercerization, LA post-mercerization showed low hydrolysis of the ether bonds between the cellulose and reactive dyes of R2 and R195 [34] (Figure 6). It is worth noting that in the CS post-mercerization of woven fabrics, the dye removal percentage (*R*%) of the R2 dye was obviously higher than that of R195, which was caused by the type of reactive group. R195 has one vinyl sulfone group and one monochrolotriazinyl group, while R2 has one dichlorotriazinyl group (Table 1). The reactive groups influence the fixed strength of the dye, whereby the R195-dyed fabrics were shown to be more stable when compared to R2-dyed fabrics after CS post-mercerization.

Moreover, after the ether bond was broken, the hydrolyzed dyes inside the fiber were easily washed out, since the CS post-mercerization enlarged the pore size of the cotton fiber, which possibly contributed to the higher *R*% of R2-dyed fabric treated by the CS post-mercerization process because the molecular size of R2 is smaller than that of R195. In addition, the CS and LA pre-mercerization processes were shown to be beneficial to the fixed R2 dye stability, especially in CS post-mercerization. The R2 *R*% of Dye-CS was 4.09%, while the same values for fabrics pre-mercerized by CS (CS-Dye-CS) and LA (LA-Dye-CS) were shown to be 3.43% and 3.86%, respectively.

### 3.5. Color Strength and Color Uniformity

**Figure 7:**
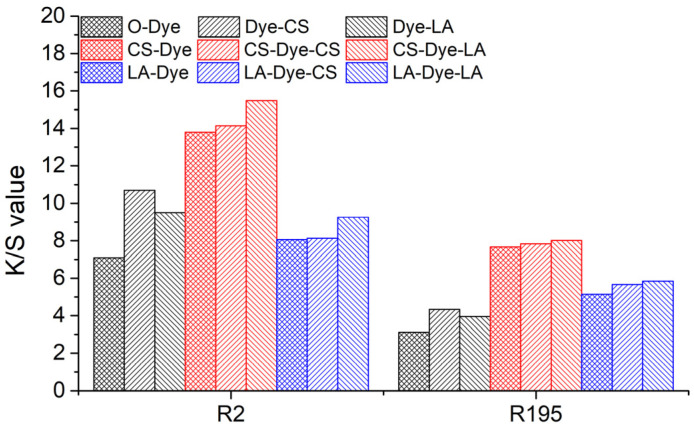
*K/S* values of the original, pre-mercerized, and post-mercerized woven cotton fabrics dyed with R2 and R195 dyes.

The *K/S* values of the original, pre-mercerized, and post-mercerized samples are shown in Figure 7. According to the Kubelka–Munk principle, the value of K/S is related to the dye concentration in the sample, i.e., the larger the value of *K/S*, the greater the dye concentration in the sample [35]. However, the *T*% values of woven fabrics dyed with R2 and R195, as well as the K/S values of these respective samples (O-Dye, CS-Dye, and LA-Dye in Table 4), indicate that this relationship is undesirable for mercerized woven cotton fabrics. The *T*% values of R2-dyed samples follow a decreasing order from CS-Dye (77.7%) to O-Dye (67.2%) and LA-Dye (60.4%), while the order of their *K/S* values is from CS-Dye (13.8) to LA-Dye (8.1) and O-Dye (7.1). This implies that the pre-mercerization obviously increased the color shade of the dyed woven cotton fabrics, owing to a decrease in light scattering of the mercerized fabrics. With the CS and LA pre-mercerization, the morphological structures of the fabrics and fibers, as well as the presence of voids, were changed, which possibly altered the irradiated light route in the fabrics and fibers. Additionally, the material features, such as the micropores of the cotton fiber and cell cavity, were probably reduced in size or eliminated. This resulted in reduced light scattering and increased color intensity [10,11].

In Table 4, based on the Kubelka–Munk principle, the *K/S* values have a linear relationship with the dye concentration in the dyed fiber. Since the *T*% of the original fiber was 67.2% with a *K/S* value of 7.1, if the *T*% values of R2 were 77.7% and 60.4% in the original woven cotton fabric, the *K/S* values for both dyed fabrics would be 8.2 (O-Dye predicted value-1) and 6.4 (O-Dye predicted value-2), respectively. However, the experimental *K/S* values of the R2-dyed fabrics with CS pre-mercerization (77.7% of *T*%) and LA pre-mercerization (60.4% of *T*%) were 13.8 and 8.1, respectively. Thus, in the R2 dye processes, considering the dye concentration increase in the dyed fabric, the promoted K/S efficiency (Equation (4)) was 68.1% for CS pre-mercerization and 26.3% for LA pre-mercerization. Additionally, the promoted K/S efficiencies in the R195 dye processes were 77.9% and 63.7% for CS-Dye and LA-Dye, respectively. A higher color strength was found in the CS pre-mercerized fabric than the LA pre-mercerized fabric. The enhanced K coefficient in the *K/S* likely accounted for the higher color strength of the CS pre-mercerized fabrics. Additionally, the light scattering was reduced by the missing cell cavity, resulting in a decrease in the S coefficient of *K/S*. The CS pre-mercerization increased the swelling of cotton fibers more than the LA pre-mercerization, which reduced the cell size of the fiber of the secondary cell wall and core [11]. Furthermore, as a result of the inner cell’s color migration to the outside cell, the color strength of the fiber was enhanced [36]. Therefore, CS pre-mercerization to the woven cotton fabric showed a more highly promoted *K/S* efficiency than the LA pre-mercerization.

As shown in Figure 7, CS and LA post-mercerization enhanced the dyed fabric *K/S* value regardless of whether the fabrics had been pre-mercerized. In the dyed fabric without pre-mercerization (O-Dye), the CS post-mercerization (Dye-CS) was more effective in promoting the *K/S* value compared to the LA post-mercerization (Dye-LA). This result is consistent with the findings of the pre-mercerization contribution to the *K/S* increase. In the CS and LA pre-mercerized and dyed fabrics (CS-Dye and LA-Dye), both CS and LA post-mercerization (CS-Dye-CS, CS-Dye-LA, LA-Dye-CS, or LA-Dye-LA) further increased the *K/S* value of the dyed fabrics, but the increases were low compared to the increases of the original dyed fabric treated by CS and LA post-mercerization process (Dye-CS and Dye-LA). Additionally, the LA post-mercerization (CS-Dye-LA and LA-Dye-LA) provided a slight increase to the *K/S* value in the CS and LA pre-mercerized and dyed fabrics compared to the CS post-mercerization process (CS-Dye-CS and LA-Dye-CS). Therefore, this indicated that the morphological structure change influenced the color shade of the cotton fabric during post-mercerization, specifically the LA post-mercerization process, which most effectively promoted an increased *K/S* value in contrast with CS post-mercerization.

**Figure 8:**
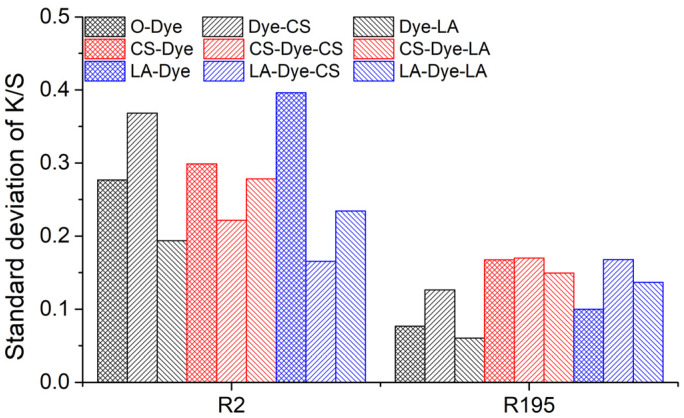
Standard deviations of *K/S* values of the original and mercerized woven cotton fabrics dyed with R2 and R195 dyes.

The color uniformities of all dyed fabrics were investigated and the results are shown in Figure 8. Notably, color uniformity was expressed by measuring the standard deviation value. All of the sample’s standard deviation values were less than 0.4, meaning that the color uniformity was very good for all dyed fabrics. This also implies that the pre-mercerization, post-mercerization, and the combination of pre- and post-mercerization processes did not cause a color unevenness problem on the dyed fabric.

### 3.6. Colorfastness to Washing and Rubbing

The colorfastness values to washing and rubbing of dyed fabrics are listed in Table 5. The wash fastness grade was evaluated by the dye stained in the cotton fiber of the tested multifiber, according to the gray scale. It was noticed that excellent wash fastness values of all samples (Grade 5) were achieved as a result of the strong covalent link formed between the cotton fiber and reactive dye. Meanwhile, the contribution from the practical washing process was significant, which was effective in washing off the unfixed dyes in the dyed fabrics. This efficient washing of the dyed fabrics was also beneficial to the rubbing fastness. In the R2-dyed fabric, the dry rubbing fastness was of grade 4–5, while grade 4 was observed for the wet rubbing fastness. In the R195 dyed fabric, the dry and wet rubbing fastness levels were grade 5 and grade 4–5, respectively. The latter values were a half grade higher for the R195-dyed fabrics than the R2-dyed fabrics in terms of dry and wet rubbing fastness.

### 3.7. Fabric Stiffness

The stiffness values of the original, pre-mercerized, and post-mercerized woven cotton fabrics dyed with R2 are listed in Table 6. The results indicate that the fabric became stiffer as a result of the combination of pre- and post-mercerization, particularly the combination of CS pre- and post-mercerization. In comparing the CS and LA pre-mercerization, the former (CS-Dye) made the fabric stiffer than the latter (LA-Dye). The total bending length and total flexural rigidity values of the fabric with LA pre-mercerization (LA-Dye) were 1.69 cm and 0.82 mg cm, respectively, which were slightly higher than the values for original sample (O-Dye) at 1.64 cm and 0.75 mg cm, respectively. The CS and LA post-mercerization processes also followed the same patterns with respect to stiffness. The CS post-mercerization still exhibited a more significant effect on stiffness enhancement than the LA post-mercerization process, with values of 2.25 cm and 1.94 mg cm for the total bending length and the total flexural rigidity of Dye-CS, respectively, compared to 1.78 cm and 0.96 mg cm for Dye-LA, respectively. Despite the pre- and post-mercerization conditions of CS or LA being the same, the stiffness distinctions were different. For the CS mercerization process, the total bending length and total flexural rigidity of the fabric with CS pre-mercerization (CS-Dye) were 1.79 cm and 0.97 mg cm, respectively, while in the fabric with CS post-mercerization (Dye-CS) the values were 2.25 cm and 1.94 mg cm, respectively. These results are similar to the findings for LA mercerization. The differences showed that the dyeing processes could weaken the stiffness enhancement resulting from the CS and LA mercerization processes. In the combination of pre- and post-mercerization, regardless of whether CS or LA pre-mercerization was used for the fabric, the stiffness was more affected by CS post-mercerization than LA post-mercerization.

### 3.8. Breaking Force and Elongation

**Figure 9:**
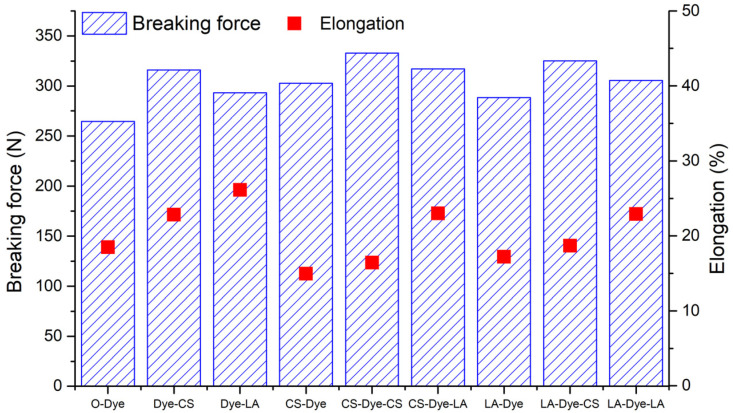
Breaking force and elongation of the original and mercerized woven cotton fabrics dyed with R2.

The breaking force and elongation at break of the woven cotton fabrics dyed with R2 are displayed in Figure 9. Generally, the pre-mercerization, post-mercerization, and combination of pre- and post-mercerization improved the breaking force of the woven cotton fabric. According to the comparison of breaking forces between CS-Dye and LA-Dye, Dye-CS and Dye-LA, and LA-Dye-CS and LA-Dye-LA, respectively, the CS mercerization was slightly more effective than the LA mercerization; however, it was exceptional in the comparison of CS-Dye-LA and CS-Dye-CS. The CS and LA mercerization swelled the fiber, reduced the convolutions from the surface, and enhanced fiber binding [27]. Additionally, the internal stresses in the yarn and fabric were released after mercerization [26]. Therefore, the weak links from the fibers, yarns, and fabric were effectively eliminated, likely due to the improved breaking force of the fabric [37]. In elongation testing, post-mercerization conferred a positive attribute to the woven cotton fabric, and the LA post-mercerization process afforded more positive contributions than the CS post-mercerization process. The CS and LA pre-mercerization processes decreased the elongation of the fabrics, but the reduction by LA pre-mercerization was lower than that by CS pre-mercerization process.

## 4. Conclusions

In this work, a caustic solution (CS) and liquid ammonia (LA) were used for pre-mercerization, post-mercerization, and a combination of pre- and post-mercerization processes on plain woven cotton fabrics. According to the results, the mercerization of cotton fabrics with CS, LA, and a combination of CS and LA changed the allomorphs of cellulose I to cellulose II, cellulose III, and a mixture of cellulose II and cellulose III, respectively. The combined mercerization process further decreased the crystallinity. Furthermore, these observations were verified by TG analysis, as the fabrics underwent faster thermal decomposition in the amorphous phase than the crystalline phase after the combined mercerization treatment. In dyeing with R2 and R195, the pre-mercerization of CS and LA increased the dye exhaustion during cotton fabric dyeing; however, the larger molecular dye (R195) showed a lower dye exhaustion rate upon dyeing of LA pre-mercerized cotton fabric. During post-mercerization, R195 exhibited higher stability and fixation with cotton cellulose. Meanwhile, the *K/S* values of the dyed cotton fabrics increased with both post-mercerization and LA post-mercerization showed higher effectiveness. The colorfastness to washing and rubbing values were satisfactory. Moreover, pre-mercerization and post-mercerization brought about higher stiffness values, and CS mercerization resulted in higher stiffness than LA mercerization. The breaking force for the CS-mercerized cotton fabric increased, while the elongation was higher for the LA-mercerized fabric. From the abovementioned results, a conclusion was made that it would be reasonable to consider a combination of pre- and post-mercerization with CS and LA to enhance the properties of woven cotton fabrics for the textile industry.

## Figures and Tables

**Table 1:** Reactive dye structures and molecular weights [16,17].

Dye	Molecular Structure	Molecular Weight (g mol^−1^)
R2	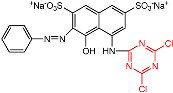	615.32
R195	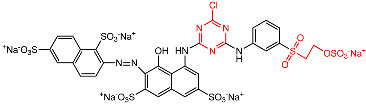	1136.28

**Table 2:** The CI values of pre-mercerized and post-mercerized cotton in LA and CS.

Sample	Original	LA	CS	LA–LA	CS–CS	LA–CS	CS–LA
*CI* (%)	73.12	52.61	63.19	51.25	58.73	38.42	40.90

**Table 3:** TG and DTG data for original and mercerized cotton fabrics.

Sample	Temperature at Initial Cleavage (T_onset_, °C)	Temperature at Maximum Degradation (T_max_, °C)	Char Residue at 700 °C (wt%)
Original	321	372	10.80
CS	319	370	10.73
LA	317	361	8.18
CS–LA	312	363	11.40
LA–CS	316	365	10.41
CS–CS	302	370	12.31
LA–LA	300	360	10.01

**Table 4:** *T*% values and *K/S* values of the original and pre-mercerized woven cotton fabrics.

Dye	Index	O-Dye	O-Dye Predicted Value-1	CS-Dye	O-Dye Predicted Value-2	LA-Dye
R2	*T*%	67.2	77.7	77.7	60.4	60.4
	*K/S*	7.1	8.2	13.8	6.4	8.1
R195	*T*%	40.3	55.5	55.5	40.5	40.5
	*K/S*	3.1	4.3	7.7	3.1	5.2

**Table 5:** Colorfastness to washing and rubbing of the original and mercerized cotton fabrics.

Sample	Wash Fastness (Grade)		Rubbing Fastness (Grade)			
	R2	R195	R2		R195	
			Dry	Wet	Dry	Wet
O-Dye	5	5	4–5	4	5	4–5
Dye-CS	5	5	4–5	4	5	4–5
Dye-LA	5	5	4–5	4	5	4–5
CS-Dye	5	5	4–5	4	5	4–5
CS-Dye-CS	5	5	4–5	4	5	4–5
CS-Dye-LA	5	5	4–5	4	5	4–5
LA-Dye	5	5	4–5	4	5	4–5
LA-Dye-CS	5	5	4–5	4	5	4–5
LA-Dye-LA	5	5	4–5	4	5	4–5

**Table 6:** Stiffness data for the original and mercerized woven cotton fabrics dyed with R2.

	O-Dye	Dye-CS	Dye-LA	CS-Dye	CS-Dye-CS	CS-Dye-LA	LA-Dye	LA-Dye-CS	LA-Dye-LA
Warp-wise bending length (cm)	1.72	2.30	1.88	2.02	2.10	2.12	1.89	2.42	2.07
Weft-wise bending length (cm)	1.57	2.21	1.69	1.58	1.96	1.77	1.51	2.02	1.71
Total bending length (cm)	1.64	2.25	1.78	1.79	2.03	1.94	1.69	2.21	1.88
Warp-wise flexural rigidity (mg·cm)	0.87	2.07	1.12	1.40	1.57	1.62	1.15	2.41	1.51
Weft-wise flexural rigidity (mg cm)	0.66	1.83	0.82	0.67	1.28	0.94	0.59	1.40	0.85
Total flexural rigidity (mg cm)	0.75	1.94	0.96	0.97	1.42	1.24	0.82	1.84	1.13

## Data Availability

The datasets generated during the current study are available from the corresponding author on reasonable request (Yingjie Cai, Y.C.).
